# 2-Methyl-5-nitro-1*H*-benzimidazole monohydrate

**DOI:** 10.1107/S1600536811019027

**Published:** 2011-05-25

**Authors:** Raza Murad Ghalib, Rokiah Hashim, Othman Sulaiman, Ching Kheng Quah, Hoong-Kun Fun

**Affiliations:** a School of Industrial Technology, Universiti Sains Malaysia, 11800 USM, Penang, Malaysia; b X-ray Crystallography Unit, School of Physics, Universiti Sains Malaysia, 11800 USM, Penang, Malaysia

## Abstract

In the title compound, C_8_H_7_N_3_O_2_·H_2_O, the 2-methyl-5-nitro-1*H*-benzimidazole mol­ecule, excluding the methyl H atoms, is approximately planar, with a maximum deviation of 0.137 (1) Å. The crystal structure is stabilized by water mol­ecules *via* N—H⋯O(water), O(water)—H⋯O and O(water)—H⋯N hydrogen bonds, forming sheets parallel to the (100) plane. A short inter­molecular contact between the benzene and imidazole rings, with a centroid–centroid distance of 3.6419 (10) Å, indicates a π–π inter­action.

## Related literature

For general background to and the potential biological activity of benzimidazole derivatives, see: Puratchikody *et al.* (2008 ▶); Tonelli *et al.* (2010 ▶); Shingalapur *et al.* (2010 ▶); Refaat (2010 ▶); Lazer *et al.* (1987 ▶). For the preparation of the title compound, see: Umare *et al.* (2008 ▶); Singh & Pathak (2008 ▶). For the stability of the temperature controller used in the data collection, see: Cosier & Glazer (1986 ▶). For standard bond-length data, see: Allen *et al.* (1987 ▶). For related structures, see: Eltayeb *et al.* (2009 ▶); Arumugam *et al.* (2010 ▶).
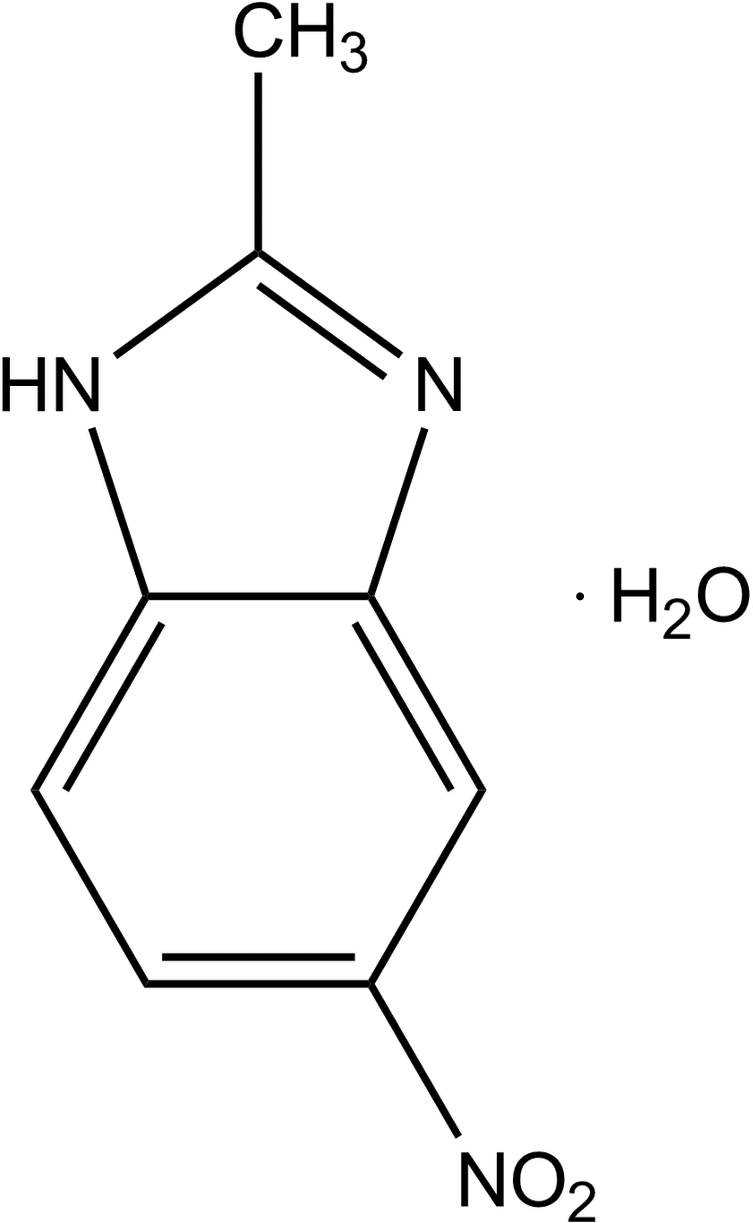


## Experimental

### 

#### Crystal data

C_8_H_7_N_3_O_2_·H_2_O
*M* *_r_* = 195.18
Triclinic, 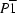

*a* = 6.9051 (10) Å
*b* = 7.1309 (11) Å
*c* = 10.0653 (15) Å
α = 79.421 (3)°
β = 73.062 (3)°
γ = 67.517 (3)°
*V* = 436.61 (11) Å^3^
*Z* = 2
Mo *K*α radiation
μ = 0.12 mm^−1^
*T* = 100 K
0.52 × 0.19 × 0.14 mm

#### Data collection

Bruker SMART APEXII DUO CCD area-detector diffractometer
Absorption correction: multi-scan (*SADABS*; Bruker, 2009 ▶) *T*_min_ = 0.942, *T*_max_ = 0.984
6312 measured reflections
1784 independent reflections
1506 reflections with *I* > 2σ(*I*)
*R*_int_ = 0.032

#### Refinement

*R*[*F* ^2^ > 2σ(*F* ^2^)] = 0.040
*wR*(*F* ^2^) = 0.115
*S* = 1.06
1784 reflections
140 parameters
H atoms treated by a mixture of independent and constrained refinement
Δρ_max_ = 0.30 e Å^−3^
Δρ_min_ = −0.35 e Å^−3^

### 

Data collection: *APEX2* (Bruker, 2009 ▶); cell refinement: *SAINT* (Bruker, 2009 ▶); data reduction: *SAINT*; program(s) used to solve structure: *SHELXTL* (Sheldrick, 2008 ▶); program(s) used to refine structure: *SHELXTL*; molecular graphics: *SHELXTL*; software used to prepare material for publication: *SHELXTL* and *PLATON* (Spek, 2009 ▶).

## Supplementary Material

Crystal structure: contains datablocks global, I. DOI: 10.1107/S1600536811019027/is2711sup1.cif

Structure factors: contains datablocks I. DOI: 10.1107/S1600536811019027/is2711Isup2.hkl

Supplementary material file. DOI: 10.1107/S1600536811019027/is2711Isup3.cml

Additional supplementary materials:  crystallographic information; 3D view; checkCIF report

## Figures and Tables

**Table 1:** Hydrogen-bond geometry (Å, °)

*D*—H⋯*A*	*D*—H	H⋯*A*	*D*⋯*A*	*D*—H⋯*A*
N1—H1*N*1⋯O1*W*^i^	0.91 (3)	1.84 (2)	2.7347 (18)	170 (2)
O1*W*—H1*W*1⋯O2^ii^	0.831 (19)	2.06 (2)	2.8737 (17)	168.5 (19)
O1*W*—H2*W*1⋯N2^iii^	0.92 (3)	1.86 (3)	2.7808 (18)	177.2 (17)

Symmetry codes: (i) 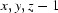; (ii) 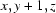; (iii) 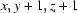.

**Fig. 1.**
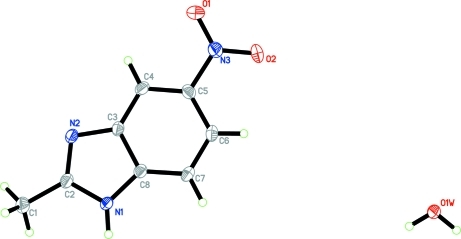
The molecular structure of the title compound showing 50% probability displacement ellipsoids for non-H atoms.

**Fig. 2.**
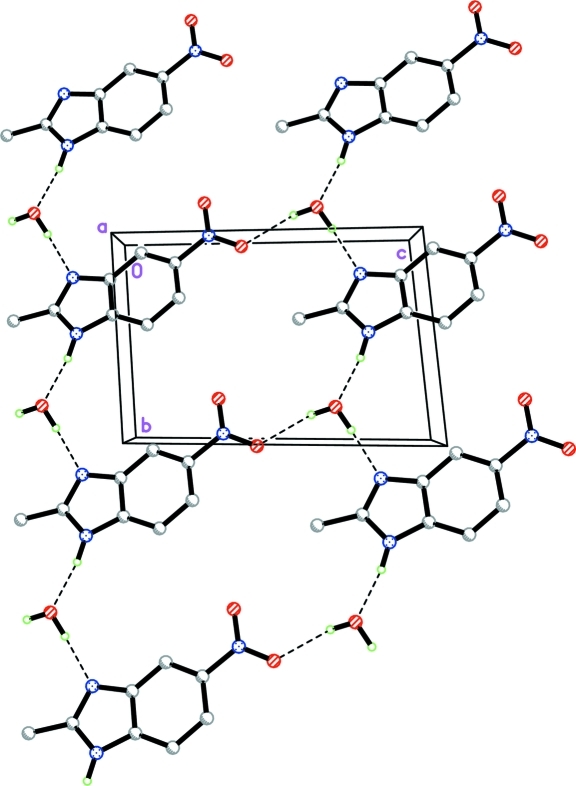
The crystal structure of the title compound, viewed along the a axis. H atoms not involved in hydrogen bonds (dashed lines) have been omitted for clarity.

## supplementary crystallographic information

### Comment

benzimidazoles are aromatic heterocylic organic compounds of wide biological importance. They are reported as antimicrobial (Puratchikody *et al.*, 2008), antiviral (Tonelli *et al.*, 2010), anticancer (Refaat, 2010), anti-inflammatory (Lazer *et al.*, 1987) and anti-diabetic (Shingalapur *et al.*, 2010) agents.

The molecular structure of the title compound is shown in Fig. 1. The 2-methyl-5-nitro-1H-benzimidazole molecule (O1/O2/N1-N3/C1-C8), excluding methyl H atoms, is almost planar, with a maximum deviation of 0.137 (1) Å for atom O2. Bond lengths (Allen *et al.*, 1987) and angles are within normal ranges and comparable to related structures (Eltayeb *et al.*, 2009; Arumugam *et al.*, 2010).

The crystal structure (Fig. 2) is stabilized by water molecules *via* intermolecular N1—H1N1···O1W, O1W—H1W1···O2 and O1W—H2W1···N2 hydrogen bonds to form sheets parallel to the (100) plane. The crystal packing is further consolidated by π–π stacking interactions between the centroids of N1/N2/C2/C3/C8 (*Cg*1) and C3–C8 (*Cg*2) rings, with *Cg*1···*Cg*2^iii^ distance of 3.6419 (10) Å [symmetry code: (iii) -*x*, 1 - *y*, -*z*].

### Experimental

4-Nitro-*o*-phenylenediamine (1.53 g) was well dissolved in acetic acid and refluxed on a heating mantle for 6 h. The reaction mixture was dried on rotavapor at low pressure to give the solid mass which was then crystallized with alcohol-chloroform (1:1 *v/v*) mixture to give the brownish crystals of title compound (Umare *et al.*, 2008; Singh & Pathak, 2008), yield 50%, *m.p.* 496-498 K. Melting point was taken on Thermo Fisher digital melting point apparatus of IA9000 series and is uncorrected.

### Refinement

O- and N-bound H atoms were located in a difference Fourier map and refined freely [O—H = 0.83 (2)–0.92 (3) Å, N1—H1N1 = 0.91 (2) Å]. The remaining H atoms were positioned geometrically and refined using a riding model with C—H = 0.93 or 0.96 Å, and with *U*_iso_(H) = 1.2 or 1.5*U*_eq_(C). A rotating-group model was applied for the methyl group. The highest residual electron density peak is located at 0.66 Å from C3 and the deepest hole is located at 0.67 Å from N3.

### Crystal data

**Table:**

C_8_H_7_N_3_O_2_·H_2_O	*Z* = 2
*M**_r_* = 195.18	*F*(000) = 204
Triclinic, *P*1	*D*_x_ = 1.485 Mg m^−^^3^
Hall symbol: -P 1	Mo *K*α radiation, λ = 0.71073 Å
*a* = 6.9051 (10) Å	Cell parameters from 2800 reflections
*b* = 7.1309 (11) Å	θ = 3.1–29.8°
*c* = 10.0653 (15) Å	µ = 0.12 mm^−^^1^
α = 79.421 (3)°	*T* = 100 K
β = 73.062 (3)°	Needle, brown
γ = 67.517 (3)°	0.52 × 0.19 × 0.14 mm
*V* = 436.61 (11) Å^3^	

### Data collection

**Table:**

Bruker SMART APEXII DUO CCD area-detector diffractometer	1784 independent reflections
Radiation source: fine-focus sealed tube	1506 reflections with *I* > 2σ(*I*)
graphite	*R*_int_ = 0.032
φ and ω scans	θ_max_ = 26.5°, θ_min_ = 2.1°
Absorption correction: multi-scan (*SADABS*; Bruker, 2009)	*h* = −8→8
*T*_min_ = 0.942, *T*_max_ = 0.984	*k* = −8→8
6312 measured reflections	*l* = −12→12

### Refinement

**Table:**

Refinement on *F*^2^	Primary atom site location: structure-invariant direct methods
Least-squares matrix: full	Secondary atom site location: difference Fourier map
*R*[*F*^2^ > 2σ(*F*^2^)] = 0.040	Hydrogen site location: inferred from neighbouring sites
*wR*(*F*^2^) = 0.115	H atoms treated by a mixture of independent and constrained refinement
*S* = 1.06	*w* = 1/[σ^2^(*F*_o_^2^) + (0.0635*P*)^2^ + 0.1355*P*] where *P* = (*F*_o_^2^ + 2*F*_c_^2^)/3
1784 reflections	(Δ/σ)_max_ = 0.001
140 parameters	Δρ_max_ = 0.30 e Å^−^^3^
0 restraints	Δρ_min_ = −0.35 e Å^−^^3^

### Special details

**Table:**

Experimental. The crystal was placed in the cold stream of an Oxford Cryosystems Cobra open-flow nitrogen cryostat (Cosier & Glazer, 1986) operating at 100.0 (1) K.
Geometry. All esds (except the esd in the dihedral angle between two l.s. planes) are estimated using the full covariance matrix. The cell esds are taken into account individually in the estimation of esds in distances, angles and torsion angles; correlations between esds in cell parameters are only used when they are defined by crystal symmetry. An approximate (isotropic) treatment of cell esds is used for estimating esds involving l.s. planes.
Refinement. Refinement of F^2^ against ALL reflections. The weighted R-factor wR and goodness of fit S are based on F^2^, conventional R-factors R are based on F, with F set to zero for negative F^2^. The threshold expression of F^2^ > 2sigma(F^2^) is used only for calculating R-factors(gt) etc. and is not relevant to the choice of reflections for refinement. R-factors based on F^2^ are statistically about twice as large as those based on F, and R- factors based on ALL data will be even larger.

### Fractional atomic coordinates and isotropic or equivalent isotropic displacement parameters (Å^2^)

**Table:**

	*x*	*y*	*z*	*U*_iso_*/*U*_eq_	
O1	0.23733 (19)	−0.19984 (17)	0.30176 (12)	0.0281 (3)	
O2	0.2283 (2)	0.0307 (2)	0.41865 (11)	0.0350 (3)	
N1	0.26105 (19)	0.47231 (19)	−0.17804 (12)	0.0176 (3)	
N2	0.2160 (2)	0.17912 (19)	−0.18388 (12)	0.0190 (3)	
N3	0.2355 (2)	−0.0294 (2)	0.30934 (13)	0.0220 (3)	
C1	0.2320 (3)	0.4036 (2)	−0.40419 (15)	0.0248 (4)	
H1A	0.1500	0.3383	−0.4285	0.037*	
H1B	0.3773	0.3594	−0.4608	0.037*	
H1C	0.1669	0.5486	−0.4198	0.037*	
C2	0.2349 (2)	0.3487 (2)	−0.25514 (15)	0.0186 (3)	
C3	0.2308 (2)	0.1917 (2)	−0.05118 (14)	0.0167 (3)	
C4	0.2203 (2)	0.0559 (2)	0.06588 (15)	0.0183 (3)	
H4A	0.1994	−0.0652	0.0652	0.022*	
C5	0.2426 (2)	0.1109 (2)	0.18391 (15)	0.0185 (3)	
C6	0.2731 (2)	0.2924 (2)	0.19023 (15)	0.0189 (3)	
H6A	0.2881	0.3206	0.2726	0.023*	
C7	0.2808 (2)	0.4286 (2)	0.07407 (15)	0.0187 (3)	
H7A	0.2995	0.5504	0.0760	0.022*	
C8	0.2594 (2)	0.3764 (2)	−0.04673 (15)	0.0168 (3)	
O1W	0.28031 (19)	0.83785 (17)	0.68930 (12)	0.0244 (3)	
H1N1	0.265 (3)	0.598 (4)	−0.212 (2)	0.036 (5)*	
H1W1	0.269 (3)	0.877 (3)	0.608 (2)	0.036 (5)*	
H2W1	0.257 (4)	0.949 (4)	0.734 (2)	0.052 (6)*	

### Atomic displacement parameters (Å^2^)

**Table:**

	*U*^11^	*U*^22^	*U*^33^	*U*^12^	*U*^13^	*U*^23^
O1	0.0388 (7)	0.0180 (6)	0.0275 (6)	−0.0098 (5)	−0.0114 (5)	0.0032 (5)
O2	0.0605 (8)	0.0352 (7)	0.0160 (6)	−0.0209 (6)	−0.0151 (5)	0.0003 (5)
N1	0.0242 (6)	0.0138 (7)	0.0168 (6)	−0.0068 (5)	−0.0075 (5)	−0.0017 (5)
N2	0.0259 (6)	0.0153 (6)	0.0177 (6)	−0.0062 (5)	−0.0089 (5)	−0.0028 (5)
N3	0.0248 (7)	0.0214 (7)	0.0194 (7)	−0.0065 (5)	−0.0076 (5)	−0.0004 (5)
C1	0.0349 (8)	0.0219 (8)	0.0186 (8)	−0.0083 (7)	−0.0104 (6)	−0.0017 (6)
C2	0.0212 (7)	0.0157 (7)	0.0191 (7)	−0.0040 (6)	−0.0069 (6)	−0.0042 (6)
C3	0.0185 (7)	0.0161 (7)	0.0162 (7)	−0.0041 (6)	−0.0064 (5)	−0.0036 (5)
C4	0.0211 (7)	0.0137 (7)	0.0211 (8)	−0.0056 (6)	−0.0062 (5)	−0.0033 (6)
C5	0.0197 (7)	0.0179 (8)	0.0163 (7)	−0.0043 (6)	−0.0061 (5)	0.0000 (6)
C6	0.0203 (7)	0.0206 (8)	0.0165 (7)	−0.0049 (6)	−0.0066 (5)	−0.0051 (6)
C7	0.0203 (7)	0.0166 (7)	0.0216 (7)	−0.0061 (6)	−0.0066 (6)	−0.0057 (6)
C8	0.0179 (7)	0.0146 (7)	0.0177 (7)	−0.0041 (5)	−0.0053 (5)	−0.0030 (5)
O1W	0.0428 (7)	0.0166 (6)	0.0191 (6)	−0.0125 (5)	−0.0127 (5)	−0.0008 (5)

### Geometric parameters (Å, °)

**Table:**

O1—N3	1.2271 (18)	C3—C4	1.384 (2)
O2—N3	1.2334 (17)	C3—C8	1.414 (2)
N1—C2	1.3660 (18)	C4—C5	1.383 (2)
N1—C8	1.3710 (19)	C4—H4A	0.9300
N1—H1N1	0.91 (2)	C5—C6	1.404 (2)
N2—C2	1.320 (2)	C6—C7	1.378 (2)
N2—C3	1.3905 (18)	C6—H6A	0.9300
N3—C5	1.4609 (19)	C7—C8	1.3973 (19)
C1—C2	1.483 (2)	C7—H7A	0.9300
C1—H1A	0.9600	O1W—H1W1	0.83 (2)
C1—H1B	0.9600	O1W—H2W1	0.92 (3)
C1—H1C	0.9600		
			
C2—N1—C8	107.18 (12)	N2—C3—C8	109.57 (13)
C2—N1—H1N1	122.1 (13)	C5—C4—C3	116.11 (13)
C8—N1—H1N1	130.4 (13)	C5—C4—H4A	121.9
C2—N2—C3	104.83 (12)	C3—C4—H4A	121.9
O1—N3—O2	123.11 (13)	C4—C5—C6	124.00 (14)
O1—N3—C5	119.20 (13)	C4—C5—N3	117.95 (13)
O2—N3—C5	117.69 (13)	C6—C5—N3	118.05 (13)
C2—C1—H1A	109.5	C7—C6—C5	119.80 (13)
C2—C1—H1B	109.5	C7—C6—H6A	120.1
H1A—C1—H1B	109.5	C5—C6—H6A	120.1
C2—C1—H1C	109.5	C6—C7—C8	117.33 (14)
H1A—C1—H1C	109.5	C6—C7—H7A	121.3
H1B—C1—H1C	109.5	C8—C7—H7A	121.3
N2—C2—N1	113.13 (13)	N1—C8—C7	132.77 (14)
N2—C2—C1	124.88 (13)	N1—C8—C3	105.30 (12)
N1—C2—C1	121.98 (13)	C7—C8—C3	121.93 (14)
C4—C3—N2	129.61 (13)	H1W1—O1W—H2W1	109 (2)
C4—C3—C8	120.82 (13)		
			
C3—N2—C2—N1	−0.04 (16)	O2—N3—C5—C6	9.3 (2)
C3—N2—C2—C1	179.09 (13)	C4—C5—C6—C7	0.4 (2)
C8—N1—C2—N2	0.05 (16)	N3—C5—C6—C7	179.98 (12)
C8—N1—C2—C1	−179.10 (13)	C5—C6—C7—C8	−0.6 (2)
C2—N2—C3—C4	179.60 (14)	C2—N1—C8—C7	179.43 (15)
C2—N2—C3—C8	0.01 (15)	C2—N1—C8—C3	−0.04 (15)
N2—C3—C4—C5	179.45 (13)	C6—C7—C8—N1	−179.40 (14)
C8—C3—C4—C5	−1.0 (2)	C6—C7—C8—C3	0.0 (2)
C3—C4—C5—C6	0.4 (2)	C4—C3—C8—N1	−179.62 (12)
C3—C4—C5—N3	−179.17 (12)	N2—C3—C8—N1	0.02 (15)
O1—N3—C5—C4	9.0 (2)	C4—C3—C8—C7	0.8 (2)
O2—N3—C5—C4	−171.14 (13)	N2—C3—C8—C7	−179.52 (12)
O1—N3—C5—C6	−170.60 (13)		

### Hydrogen-bond geometry (Å, °)

**Table:**

*D*—H···*A*	*D*—H	H···*A*	*D*···*A*	*D*—H···*A*
N1—H1N1···O1W^i^	0.91 (3)	1.84 (2)	2.7347 (18)	170 (2)
O1W—H1W1···O2^ii^	0.831 (19)	2.06 (2)	2.8737 (17)	168.5 (19)
O1W—H2W1···N2^iii^	0.92 (3)	1.86 (3)	2.7808 (18)	177.2 (17)

Symmetry codes: (i) *x*, *y*, *z*−1; (ii) *x*, *y*+1, *z*; (iii) *x*, *y*+1, *z*+1.
